# Development and validation of a prediction model to assess the probability of tuberculous pleural effusion in patients with unexplained pleural effusion

**DOI:** 10.1038/s41598-023-38048-2

**Published:** 2023-07-05

**Authors:** Xiaoli Lei, Junli Wang, Zhigang Yang

**Affiliations:** 1 Department of Pulmonary and Critical Care Medicine, Henan Provincial People’s Hospital, People’s Hospital of Zhengzhou University, Zhengzhou, 450003 Henan China; 2 Department of Cardiopulmonary Function, Fuwai Central China Cardiovascular Hospital, Zhengzhou, 451464 Henan China

**Keywords:** Risk factors, Experimental models of disease

## Abstract

Differentiating tuberculous pleural effusion (TPE) from non-tuberculosis pleural effusion remains a challenge in clinical practice. This study aimed to develop and externally validate a novel prediction model using the peripheral blood tuberculous infection of T cells spot test (T-SPOT.TB) to assess the probability of TPE in patients with unexplained pleural effusion. Patients with pleural effusion and confirmed etiology were included in this study. A retrospective derivation population was used to develop and internally validate the predictive model. Clinical, radiological, and laboratory features were collected, and important predictors were selected using the least absolute shrinkage and selection operator. The prediction model, presented as a web calculator, was developed using multivariable logistic regression. The predictive performance of the model was evaluated for discrimination and calibration and verified in an independent validation population. The developed prediction model included age, positive T-SPOT.TB result, logarithm of the ratio of mononuclear cells to multiple nuclear cells in pleural effusion (lnRMMPE), and adenosine deaminase in pleural effusion ≥ 40 U/L. The model demonstrated good discrimination [with area under the curve of 0.837 and 0.903, respectively] and calibration (with a Brier score of 0.159 and 0.119, respectively) in both the derivation population and the validation population. Using a cutoff value of 60%, the sensitivity and specificity for identifying TPE were 70% and 88%, respectively, in the derivation population, and 77% and 92%, respectively, in the validation population. A novel predictive model based on T-SPOT.TB was developed and externally validated, demonstrating good diagnostic performance in identifying TPE.

## Introduction

Tuberculous pleural effusion (TPE), characterized by a large amount of chronic effusion and inflammatory cell accumulation in the pleural cavity, is caused by *Mycobacterium tuberculosis* infecting the pleura^1^. It is a common cause of pleural effusion (PE) in tuberculosis epidemic areas^1^. So far, the diagnosis of TPE is rather difficult. The accepted gold standard for the diagnosis of TPE is that the diagnosis can be confirmed if *Mycobacterium tuberculosis* is detected in PE or pleura tissue specimen^2,3^. However, the detection rate of PE microorganism culture is low and the culture time is long (up to 8 weeks). In addition, the acquisition of pleura is an invasive procedure whether through transthoracic needle pleural biopsy or thoracoscopic pleural biopsy^4,5^. In addition, if TPE is not treated as soon as possible, some patients often develop into active tuberculosis later^1^. Therefore, there is an urgent need to develop an accurate, simple and safe diagnostic method.

In recent years, some clinical prediction models have been proposed to accurately diagnose TPE^6–8^. However, due to differences in the source of research objects, variables, and model construction methods, the clinical models proposed by different studies to diagnose TPE vary greatly. In addition, with the progress of diagnostic methods, tuberculous infection of T cells spot test (T-SPOT.TB) have been widely used in the diagnosis of tuberculosis^2,3^. However, only few studies that included T-SPOT.TB in TPE diagnostic model have been reported to date. Therefore, we developed and validated a clinical prediction model based on peripheral blood T-SPOT.TB, hoping to provide clinicians a tool that could predict the probability of TPE accurately, simply and safely.

## Materials and methods

### Derivation population for developing prediction model

We retrospectively collected clinical data of all inpatients with pleural effusion of unknown cause who underwent thoracoscopic examination in Henan Provincial People's Hospital from January 1, 2014 to September 25, 2019. Henan Provincial People's Hospital is a large teaching hospital located in Zhengzhou, China. Only patients meeting all the following conditions were eligible to participate in the study: (1) patients with pleural effusion of unknown cause at hospital admission; (2) patients who underwent thoracoscopic examination within 2 weeks of hospitalization; (3) patients who were ≥ 18 years at the time of admission. The exclusion criteria were as follows: (1) patients with neither pleural effusion dection results nor T-SPOT.TB results; (2) patients without T-SPOT.TB results only; (3) patients without pleural effusion detection results only; (4) patients with undetermined cause of pleural effusion. Patients without pleural effusion detection results refers to patients who had undergone thoracoscopy but had not undergone pleural effusion examination.

This study was approved by the ethics committee of Henan Provincial People's Hospital. Written informed consent was waived owing to the use of retrospective data. All methods were performed in accordance with the relevant guidelines and regulations.

### Population used for external validation

To assess external validation of the prediction model, we retrospectively used an independent population which included all inpatients who were admitted to Henan Provincial People's Hospital for thoracoscopic examination due to unexplained pleural effusion from September 26, 2019 to August 4, 2022. The inclusion criteria and exclusion criteria of the validation population were the same as those of the derivation population.

### Data collection

Whether in the derivation population or in the validation population, the researchers used structured data tables customized for this study to collect clinical data of qualified patients. These clinical data were obtained from the discharged medical records of patients. Two experienced respiratory doctors reviewed, refined and cross checked the patient's clinical data. All data were collected by blinding data collectors to the final outcome measures.

### Outcome measures

The outcome categories were TPE and non-tuberculous pleural effusion (non-TPE). Diagnostic criteria of TPE were as follows: (1) Ziehl–Neelsen staining or Lowenstein-Jensen cultures positive of PE, or pleural biopsy specimens; (2) Histopathology of pleural biopsy revealed granuloma^1^. Thoracoscopy must be completed within 2 weeks after admission. Thoracoscopic operation was carried out according to the method recommended in the literature^1^. Non-TPE included parapneumonic pleural effusion, empyema, malignant pleural effusion and pleural effusion caused by other reasons. If some specific malignant tumor cells were found by the pathological cytology of PE or pleural biopsy specimen, the diagnosis of a malignant tumor could be determined^9^. The diagnosis of other diseases causing PE followed the corresponding clinical diagnostic criteria^9^.

### Predictive variables

Based upon key literatures on predictive models of TPE and our clinical experience, we selected 15 candidate variables, which were not only clinically available but also minimally invasive^6–8^. Those potential predictive variables included the following clinical characteristics of patients: demographic data, medical history, symptoms, imaging results and laboratory findings. Whereas demographic variables included age and sex, medical history included the history of diabetes and the history of glucocorticoid use. Similarly, clinical symptoms included fever, cough, chest pain and chest distress. Imaging examination included the presence of abnormal lesion in the lungs and the location of pleural effusion. Laboratory findings included peripheral blood T-SPOT.TB, and routine biochemical examination of PE such as ratio of mononuclear cells to multiple nuclear cells (RMMPE), ratio of total protein in PE to total protein in serum (TPPE/TPS), ratio of LDH in PE to LDH in serum (LDHPE/LDHS) and ADA in PE (ADAPE). The source of the specimen is peripheral blood or pleural effusion or pleura obtained during the patient's hospitalization. The peripheral blood T-SPOT.TB test was performed in accordance with the instructions of the T-SPOT.TB kit (Oxford Immunotec, UK)^10^. For RMMPE, we used a logarithmic transformation (lnRMMPE).We tested the linearity of continuous variables with restricted cubic splines using the Hmisc and Design library in R statistical software^11,12^. A linear relationship with the outcomes was found to be a good approximation for age and lnRMMPE. According to the Light's criteria, TPPE/TPS, LDHPE/LDHS and ADAPE, which had no linear relationship with the outcomes, were converted to dichotomous data with generally accepted cut-off values of 0.5, 0.67 and 40 U/L, respectively^1^.

### Modelling procedure and validation

Fifteen variables in the derivation population were included for variable selection and development of prediction model. Variables identified by least absolute shrinkage and selection operator (LASSO) regression analysis were entered into logistic regression model. Enhanced bootstrap method was applied to validate the developed prediction model internally. The prediction model was externally validated using validation population. Data were analyzed between September 1, 2022 and October 31, 2022.

### Model performance

The predictive performance of the prediction model was estimated by discrimination and calibration. The area under the receiver operator characteristic curve (AUC) was used to estimate the ability to discriminate of the prediction model. The diagnostic performance (sensitivity, specificity, positive and negative predictive values, and positive and negative likelihood ratios) for several risk thresholds (from 40 to 80%) of patients with TPE compared with non-TPE were calculated. The Youden index was used to determine the cutoff value for the risk probability of TPE, because at the highest level of the Youden index, the cutoff value has the highest sensitivity and specificity for diagnosing TPE. To assess calibration of prediction model, we compared the predicted probability of TPE with the observed proportions of TPE. Calibration curve was constructed, and Brier score was calculated by “rms” package in R statistical software. Brier score is an indicator to evaluate the calibration, and if it is less than 0.25, it usually indicates that the calibration of a prediction model is good.

### Presentation of the prediction model

A digital calculator was constructed to illustrate and accommodate potential clinical use. The calculator was based directly on the final regression formula, which was available at the website (https://yzg1972.shinyapps.io/dynnomapp/?_ga=2.167916065.1853486038.1670763324-35843408.1670299435).

### Statistical analysis

For the consideration of sample size, we followed the principle that the effective sample size was greater than 10 times the number of prediction variables. Variables with > 20% missing data were excluded. Imputation for missing variables was considered if missing values were less than 20%. We used predictive median matching to impute numeric variables. The “glmnet” package was used to perform the LASSO regression. A multivariable prediction model was developed by using ‘lrm’ function in R ‘rms’ package. Continuous variables were presented as median (interquartile ranges, IQR), and qualitative data were presented using frequency distribution n (%). SPSS software v.23.0 (IBM Inc., Chicago, IL, USA) and R software v.4.2.1 (R Core Team (2022). R: A language and environment for statistical computing. R Foundation for Statistical Computing, Vienna, Austria. URL https://www.R-project.org/)were used for statistical analysis. All significance tests were two-tailed, and *P* < 0.05 was considered as statistically significant.

### Ethics approval and consent to participate

The study was approved by Henan Provincial People’s Hospital Medical Ethics Committee (No.2020119), and the requirement for written informed consent was waived by the Henan Provincial People’s Hospital Medical Ethics Committee.

## Results

### Characteristics and outcome measures of derivation population and validation population

**Figure 1:**
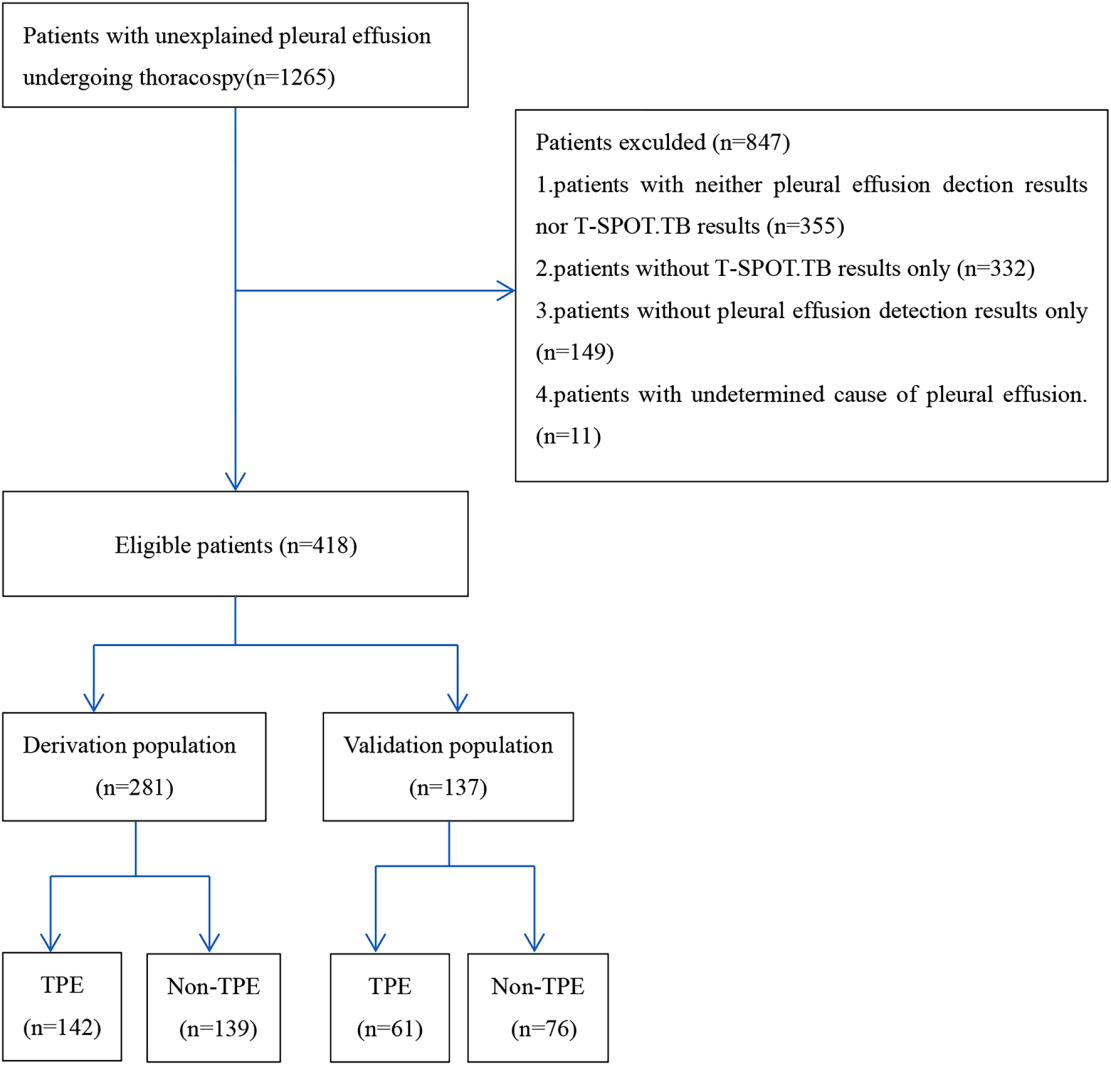
Flowchart of the study participants.

The flowchart of the patient inclusion and exclusion process in this study is presented in Fig. 1. A total of 418 eligible patients were reviewed and divided into the derivation populations (n = 281) and the external validation populations (n = 137). The general characteristics of the populations and the validation population are summarized in Table 1. A similar prevalence of TPE was observed in both the derivation population (50.5%) and validation population (44.5%). Moreover, the two population groups exhibited slight difference for some characteristics such as age and sex. Univariate logistic regression analysis of TPE diagnosis in the derivation population is shown in Table 2.

**Table 1:** General characteristics and outcome measures of derivation and validation populations. Values are numbers (percentages) unless stated otherwise.

Variables	Derivation population (n = 281)	Validation population (n = 137)
Age, median [IQR], years	56 (40–69)	58 (43–69)
Male	202 (72%)	101 (74%)
T-SPOT.TB ( +)	185 (66%)	78 (57%)
lnRMMPE, median [IQR]	2.14 (0.18–3.21)	1.93 (0.69–3.20)
ADAPE ≥ 40U/L	87 (31%)	48 (35%)
Outcome measures		
TPE	142 (50.5%)	61 (44.5%)
Culture-confirmed	9 (6.3%)	5 (8.2%)
Biopsy-compatible	133 (93.7%)	56 (91.8%)
Non-TPE	139 (49.5%)	76 (55.5%)
Parapneumonic effusion	29 (10.3%)	13 (9.5%)
Empyema	18 (6.4%)	9 (6.6%)
Bacterial pleural effusion	19 (6.8%)	15 (10.9%)
Connective tissue disease	2 (0.7%)	3 (2.2%)
Other aseptic inflammation	1 (0.4%)	5 (3.6%)
Lung cancer	38 (13.5%)	23 (16.8%)
Malignant mesothelioma	14 (5.0%)	3 (2.2%)
Other types of tumors	13 (4.6%)	3 (2.2%)
Transudate	5 (1.8%)	2 (1.5%)

*IQR* interquartile range, *TPE* tuberculosis pleural effusion, *Non-TPE* non tuberculosis pleural effusion, *T-SPOT.TB* tuberculous infection of T cells spot test, *lnRMMPE* logarithm of the ratio of mononuclear cells to multiple nuclear cells in pleural effusion, *ADAPE* adenosine deaminase in pleural effusion.

**Table 2:** Univariate logistic regression analysis of TPE diagnosis in the derivation population.

Variables	OR	(95% CI)	*P* value
Age, year	0.96	(0.94–0.98)	< 0.001
Male	0.94	(0.46–1.90)	0.9
Fever	1.97	(1.00–3.91)	0.05
Cough	0.94	(0.49–1.81)	0.9
Chest pain	0.95	(0.48–1.87)	0.9
Chest distress	2.02	(0.99–4.24)	0.057
Diabetes mellitus	0.36	(0.13–0.95)	0.044
Glucocorticoid application	0.37	(0.03–2.84)	0.4
Abnormal lesions of chest imaging	0.75	(0.36–1.55)	0.4
Location of pleural effusion			
Right side	Ref		
Left side	0.63	(0.30–1.28)	0.2
Both sides	1.2	(0.50–2.91)	0.7
T-SPOT.TB ( +)	4.79	(2.35–10.1)	< 0.001
lnRMMPE	1.4	(1.18–1.69)	< 0.001
TPPE/TPS ≥ 0.5	3.7	(1.13–14.6)	0.043
LDHPE/LDHS ≥ 0.67	0.94	(0.23–4.13)	> 0.9
ADAPE ≥ 40U/L	2.57	(1.21–5.64)	0.016

Among 281 subjects in the derivation population, 142 (50.5%) were diagnosed with TPE and 139 (49.5%) were diagnosed with non-TPE. Among 137 subjects in the validation population, 61 (44.5%) were diagnosed with TPE and 76 (55.5%) were diagnosed with non-TPE. Among patients diagnosed with TPE, the number of culture-confirmed cases in the derivation and validation population was 9 (6.3%) and 5 (8.2%), respectively. The number of biopsy-compatible cases was 133 (93.7%) and 56 (91.8%), respectively (Table 1).

### Model development and performance

**Figure 2:**
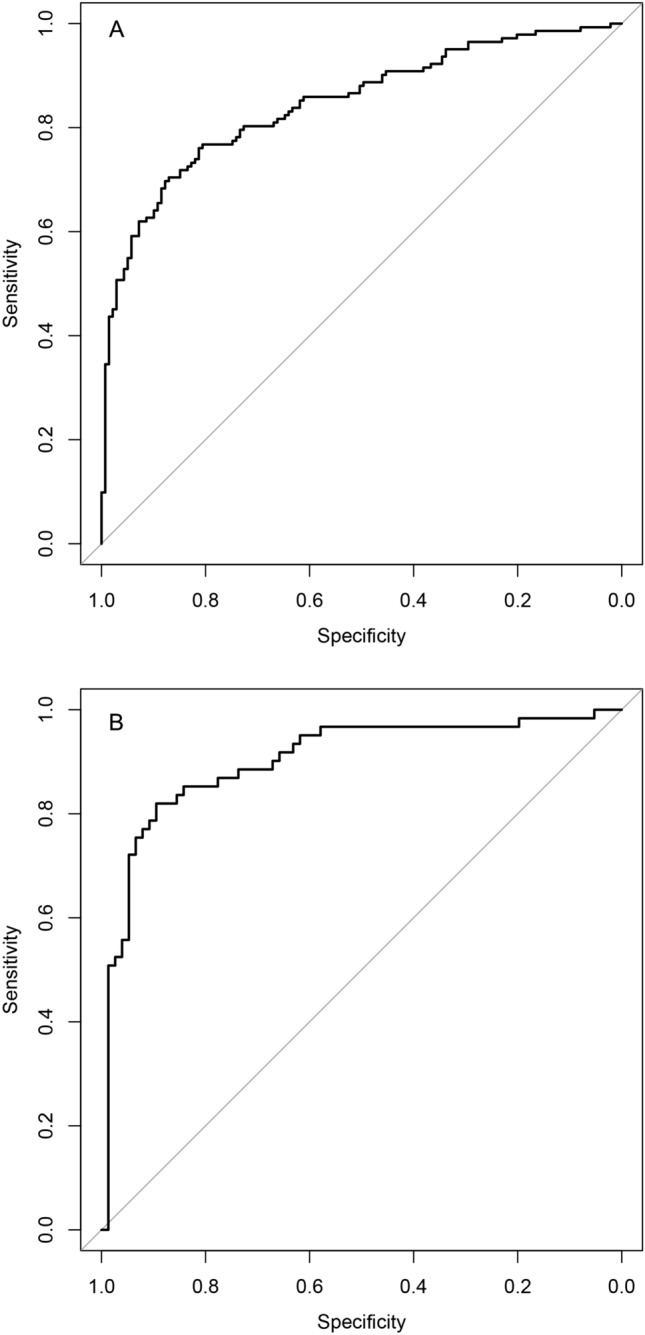
Receiver operating characteristic curve for TPE diagnosis in derivation population (**A**) and in the validation population (**B**). *T-SPOT.TB* tuberculous infection of T cells spot test, *lnRMMPE* logarithm of the ratio of mononuclear cells to multiple nuclear cells in pleural effusion, *ADAPE* adenosine deaminase in pleural effusion.

**Figure 3:**
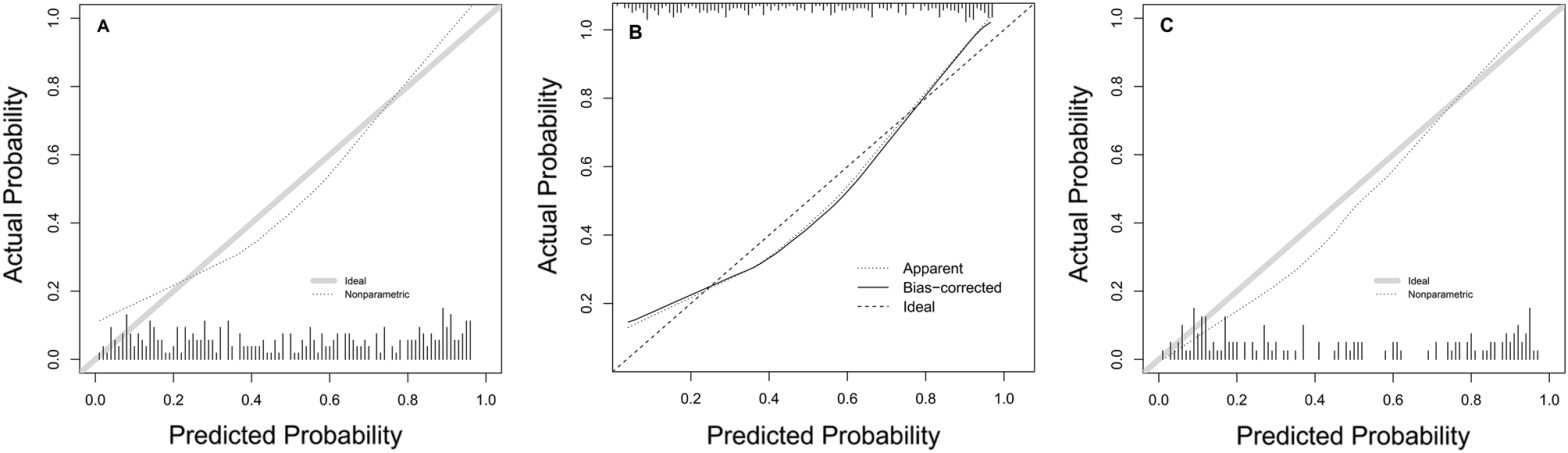
Calibration plot of the prediction of TPE probability in the derivation population (**A**), in internal validation (**B**) and in the external validation population (**C**). In figures (**A,C**), the solid diagonal line represents ideal calibration and dotted line is the calibration line of the prediction model. In figure (**B**), dashed diagonal line represents ideal calibration, straight line represents the bias-corrected calibration line of the prediction model, and dotted line represents the apparent calibration line of the prediction model.

In the derivation population, 15 variables were first included in the LASSO regression (Supplementary Fig. 1). After LASSO regression selection, 4 variables were selected as significant predictors for diagnosing TPE, which were then included in the logistic regression model. These four predictors of TPE were age per year (odds ratio 0.96, 95% CI 0.94 to 0.98 per year), positive T-SPOT.TB result (5.04, 2.63 to 10.0), lnRMMPE (1.40, 1.20 to 1.66) and ADAPE ≥ 40 U/L (2.87, 1.45 to 5.82). Table 3 presents multivariate logistic regression analysis of TPE diagnosis in the derivation population. In the derivation population the AUC for TPE diagnosis was 0.845 (95% CI 0.800–0.891) (Fig. 2A) and Brier score was 0.159. Figure 3A shows calibration plot of the prediction of TPE diagnosis in the derivation population. The AUC for TPE diagnosis was 0.837 by internal bootstrap validation. Figure 3B shows that the bias-corrected calibration curve roughly overlaps with the ideal reference curve, indicating that the model still has good calibration after internal verification.

**Table 3:** Multivariate logistic regression analysis of TPE diagnosis in the derivation population.

Variables	OR	(95% CI)	*P* value
Age, year	0.96	(0.94–0.98)	< 0.001
T-SPOT.TB ( +)	5.04	(2.63–10.0)	< 0.001
lnRMMPE	1.4	(1.20–1.66)	< 0.001
ADAPE ≥ 40U/L	2.87	(1.45–5.82)	0.003

In the derivation population, low risk thresholds were useful to rule out the diagnosis of TPE. A risk cut-off of 40% had a sensitivity of 0.81 (95% CI 0.74 to 0.87) and a negative likelihood ratio of 0.28. According to the ROC, the probability of 60% is selected as the cut off value of TPE diagnosis because Youden index is the maximum at this level. Risk thresholds of 60% or more were useful to identify TPE. For example, a risk threshold of 70% had a specificity of 0.94 (0.89–0.98) and positive likelihood ratio of 10.28 (Table 4). Figure 2A shows the ROC for the prediction of TPE in the derivation population.

**Table 4:** Diagnostic performance measures at different risk thresholds of the prediction model in derivation population and validation population.

Group	Indicators	Risk threshod				
		> 40%	> 50%	> 60%	> 70%	> 80%
Derivation population	Sensitivity (95%CI)	0.81 (0.74–0.87)	0.77 (0.71–0.83)	0.70 (0.62–0.77)	0.59 (0.51–0.67)	0.45 (0.37–0.54)
	Specificity (95%CI)	0.67 (0.58–0.75)	0.75 (0.67–0.82)	0.88 (0.81–0.93)	0.94 (0.89–0.98)	0.94 (0.93–0.99)
	+ LR	2.45	3.05	5.7	10.28	15.66
	− LR	0.28	0.31	0.35	0.43	0.57
	+ PV(%)	71.4	75.7	85.3	91.3	94.1
	− PV(%)	77.5	75.9	73.9	69.3	63.4
Validation population	Sensitivity (95%CI)	0.85 (0.74–0.93)	0.82 (0.70–0.91)	0.77 (0.65–0.87)	0.72 (0.59–0.83)	0.56 (0.42–0.69)
	Specificity (95%CI)	0.78 (0.67–0.86)	0.86 (0.76–0.93)	0.92 (0.84–0.97)	0.95 (0.87–0.99)	0.96 (0.89–0.99)
	+ LR	3.81	5.66	9.76	13.7	14.12
	− LR	0.19	0.21	0.25	0.29	0.46
	+ PV(%)	75.4	82	88.7	91.7	91.9
	− PV(%)	86.8	85.5	83.3	80.9	73.0

+ *LR* positive likelihood ratio, − *LR* negative likelihood ratio, + *PV* positive predictive value, − *PV* negative predictive value.

The prediction model for TPE diagnosis in patients with unknown effusion expresses the probability of TPE as a function of the 4 clinical variables as follows:

1. Probability of TPE = e^x/(1 + e^x)
2. x = 0.3483 − (0.0424 × Age) + (1.6172 × T-SPOT.TB) + (0.3397 × lnRMMPE) + (1.0530 × ADAPE)

where e is the base of natural logarithms; age is the patient's age in years; T-SPOT.TB = 1 if the result of the patient's T-SPOT.TB test is positive and 0 if otherwise; lnRMMPE is logarithmic transformation of RMMPE; ADAPE = 1 if patient's ADAPE concentration is equal to or greater than 40 U/L and 0 if otherwise.

**Figure 4:**
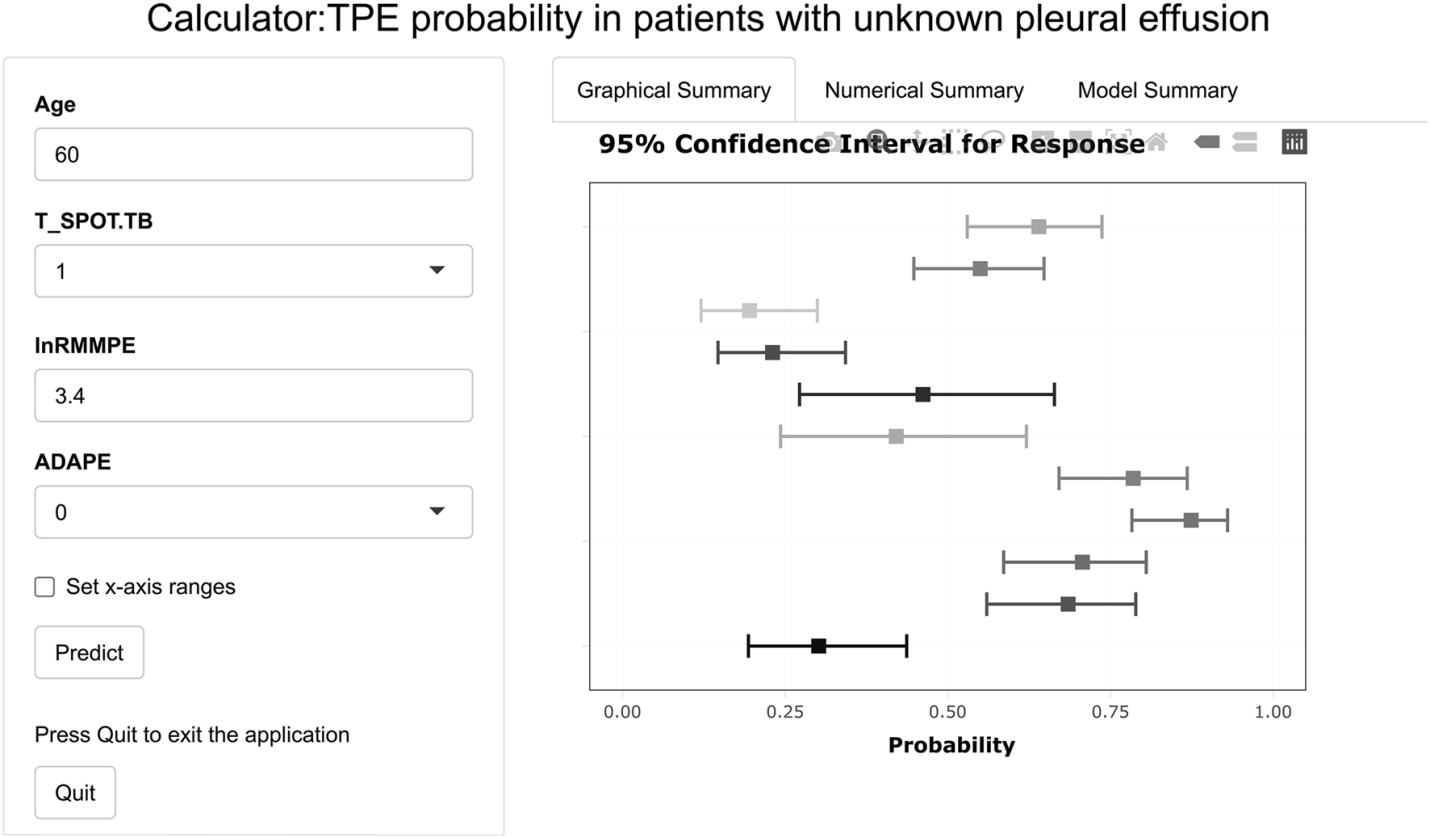
Calculator: TPE probability in patients with unknown pleural effusion. *Note*: “Age” is the patient's age in years; “T_SPOT.TB” = 1 if the patient's T-SPOT.TB result is positive and 0 if otherwise; “lnRMMPE” is the logarithm of the ratio of mononuclear cells to multinuclear cells in patient's pleural effusion; “ADAPE” = 1 if the patient's pleural effusion ADA is ≥ 40 U/L and 0 if otherwise. The calculator can be available at the website: https://yzg1972.shinyapps.io/dynnomapp/?_ga=2.167916065.1853486038.1670763324-35843408.1670299435.

An online calculator was developed to allow clinicians to enter the values of the four required variables with automatic calculation of the probability of TPE in patients with unknown effusion (Fig. 4).

### External validation of prediction model

In the external validation population the AUC of this model to predict the presence of TPE was 0.903 (0.847–0.958). Figure 2B shows the ROC for the prediction of TPE diagnosis in the validation population. Calibration for the internal validation was adequate for outcome and Brier value was 0.119. Figure 3C shows that the calibration curve of the prediction model roughly overlaps with the ideal calibration curve in the validation population.

In the validation population, low risk thresholds could also rule out the presence of TPE. For example, a risk threshold of 40% or more had a sensitivity of 0.85 (0.74–0.93) and negative likelihood ratio of 0.19. High risk thresholds of 60% or more were useful to identify TPE. For example, a risk threshold of 70% had a specificity of 0.95 (0.87–0.99) and positive likelihood ratio of 13.7 (Table 4).

ROC analysis demonstrated that the AUC for the developed multi-parameter prediction model, as well as for the individual application of T-SPOT.TB, ADA, lymphocyte/neutrophil ratio, and the combined use of ADA and lymphocyte/neutrophil ratio in diagnosing TPE, were as follows: 0.845 (95% CI 0.800–0.891), 0.710 (0.649–0.772), 0.771 (0.712–0.830), 0.691 (0.629–0.753), and 0.726 (0.667–0.784), respectively. Hence, the discrimination ability of our developed multi-parameter prediction model for TPE outperformed other parameters (all *p* < 0.05, compared to the predictive model). For more details, refer to Supplementary Table 1 and Fig. 2.

## Discussion

In this study, we developed and externally validated an accurate, simple, reliable and minimally invasive prediction model for the probability of TPE in patients with unknown pleural effusion. The model was shown in the form of web calculator convenient for clinicians, and contained four strong predictors of TPE: age, positive T-SPOT.TB, lnRMMPE and ADAPE ≥ 40 U/L. High risk thresholds of 60% or more were able to rule in TPE diagnosis.

Owing to the difficulty of TPE diagnosis, different scholars have been attempting to apply various available non-invasive variables to develop multiple prediction models for TPE diagnosis. However, the conclusions of previous studies were different and the studies were associated with pertinent limitations which impacts their applications. For example, Porcel et al. developed two clinical scoring models for differentiating TPE and malignant PE^13^. The variables included in model 1 were ADA > 40 U/L, age < 35 years, temperature > 37.8 °C, red blood cell count < 5 × 10^9^/L. In model 2, the included variables were age < 35 years, temperature > 37.8 °C, red blood cell count < 5 × 10^9^/L, no previous history of malignant tumors and pleural effusion/serum LDH ratio > 2.2. The sensitivity of model 1 and model 2 was 95% and 97%, respectively. Whereas the specificity was 94% and 91% for model 1 and model 2, respectively. The model was limited to the identification of TPE and malignant PE, and excludes other common etiologies, such as infectious diseases or rheumatic diseases, which may also lead to PE. Our study included various etiologies of common non-TPE in clinic, and thus not limited to merely distinguishing TPE from malignant PE. Sun et al. also proposed a diagnostic scoring model for TPE. A total of seven factors were included in the model, which were body temperature > 38 °C (1 point), positive tuberculin skin test (1 point), C-reactive protein ≥ 26 mg/L (1.5 points), PE lymphocyte ratio ≥ 85% (1 point), PE protein ≥ 49 g/L (1 point), ADAPE ≥ 43 U/L (2.5 points), and any positive tuberculosis antibody in blood and PE (2 points). When the score is ≥ 6.0, the sensitivity, specificity and accuracy of the model in diagnosing TPE were 90.1%, 94.3% and 92.3%, respectively^14^. Although some of the variables selected in this study such as PE lymphocyte ratio and PEADA were similar to ours, the variables included in the model were not convenient for clinical application. Moreover, due to the high prevalence of tuberculosis and high vaccination rate of BCG in China, the specificity of tuberculin skin test is too low, which led to the examination being stopped in most hospitals. Demirer et al. developed a cheap and simple prediction model, which only included two variables (age < 47 years and PEADA > 35 U/L)^7^. The variables of this study were essentially similar to that of our study. However, their study was not without a drawback as it did not include interferon-γ release assays (IGRAs) data which discriminate TPE from non-TPE, and the diagnostic performance of their model was not verified in a prospective independent cohort^7^.

Our study revealed that positive T-SPOT.TB in peripheral blood was independently associated with the diagnosis of TPE. Therefore, it was selected as an important predictive variable with diagnostic value. T-SPOT.TB, as one of commercial products of IGRAs developed in recent years, has been widely used in the diagnosis of latent tuberculosis^2,3^. Losi et al^15^ found that the sensitivity and specificity of T-SPOT.TB in peripheral blood in the diagnosis of TPE were 90% and 67%, respectively. However, a similar study found that the sensitivity and specificity of peripheral blood T-SPOT.TB in the diagnosis of TPE were only 73% and 73.1%, respectively^16^. It can be seen that there were great differences in the sensitivity and specificity of T-SPOT.TB in the diagnosis of TPE when applied separately, especially the low specificity, which can easily cause misdiagnosis of TPE. Thus, the use of T-SPOT.TB in the diagnosis of TPE needs to be studied in the future. Moreover, studies on prediction model with T-SPOT.TB as the predictor are relatively few. Only one study reported in 2020 showed that the predictive nomogram included TB-IGRA is superior to the single TB-IGRA detection in diagnosing TPE. The confirmed tuberculous pleurisy and presumptive tuberculous pleurisy nomograms had an AUC of 0.93 (95% CI 0.90–0.95) and 0.92 (95% CI 0.90–0.94) in the training group, and 0.91 (95% CI 0.87–0.96) and 0.93 (95% CI 0.89–0.96) in the validation group, respectively^17^. However, the IGRAs used in mentioned research was to measure the amount of released interferon-γ, rather than the more commonly used T-SPOT.TB method. Additionally, the model had 12 predictors, which was inconvenient for clinical use, and the calibration of the model was not analyzed. Cognizant of this fact, the prediction model proposed in this study included four commonly used clinical variables including T-SPOT.TB. The AUC of the model for diagnosing TPE was 0.845 (95% CI 0.800–0.891), which greatly improved the prediction performance of TPE diagnosis.

Similar to previous studies, our study found that elevated ADA in PE was independently correlated with the diagnosis of TPE. It was considered that PEADA < 40 U/L was rarely caused by tuberculosis, and it was an excellent examination to exclude tuberculosis^18^. The higher the ADA level, the greater the probability of TPE, while the lower the ADA level, the lower the probability of TPE^19^. Therefore, the increase of ADA in PE was naturally reasonably included in our model.

The study showed that in 60%-90% of TPE, the cells in pleural effusion are mainly infiltrated by lymphocytes, and the other cases are mainly neutrophils^20^. While the multinuclear neutrophils predominate in the first few days after the onset of tuberculous pleurisy, the mononuclear lymphocytes predominate thereafter^21^. We previously found that pleural effusion mononuclear cells count is relatively useful for TPE diagnosis^22^. Another study also revealed that mononuclear cell/leukocyte ratio was significantly higher in TPE than in non-TPE^23^. On the basis of the these studies, in this study lnRMMPE is found to be an additional important predictor of TPE, which indirectly confirmed the important diagnostic value of lymphocytes in pleural effusion for TPE.

An epidemiological study from the United States showed that about 50% of TPE patients were under 45 years old^24^. In areas with high burden of tuberculosis, TPE mainly affects young people (average age = 34 years), of which primary infection accounts for a large percentage of patients with TPE^13^. Our findings are similar to these studies. We found that age was an independent predictor of TPE diagnosis and was negatively related to the diagnosis of TPE (OR 0.96; 95% CI 0.94–0.98; *P* < 0.001).

Our study had some advantages. Firstly, all the patients included in this study underwent thoracoscopy, and the pleural specimens obtained were examined by histopathology and acid fast staining. The etiological diagnosis of TPE and non-TPE was accurate, making the bias from the gold standard small. Secondly, the developed prediction model in derivation population was verified in an independent validation population to confirm the reliability of the prediction performance of the model. Thirdly, the four variables included in the model were easy to obtain and minimally invasive, so the model was more simple, practical and safe. Finally, we first included T-SPOT.TB in the diagnosis model of TPE, which further proved the diagnostic value of T-SPOT.TB for TPE.

Inevitably, this study also had some limitations. Firstly, the collected data were clinically driven rather than research driven, and case selection bias is difficult to avoid. Therefore, the conclusions of this study needed to be interpreted carefully when applied clinically. Secondly, this study was a single center study. The research unit is a university affiliated hospital. The cases might not truly represent the overall TPE population. Therefore, the population to which the conclusions of this study are applied should be similar to the population included in this study as much as possible. For example, in order to increase the accuracy of the study, we used live tissue samples obtained through thoracoscopy for histopathological examination as the gold standard for diagnosis. Of course, this was not without a cost, which was that patients who received thoracoscopy were usually younger. Therefore, our findings may not be suitable for patients who cannot tolerate thoracoscopy, such as physically weak patients or elderly patients.Thirdly, this study was a retrospective study. Thus, prospective and multicenter studies are needed to further verify the conclusion of this study. One final disadvantage to note was that the T-SPOT.TB result was an important variable that constituted our model. In nations where tuberculosis is prevalent, particularly in Asia, the likelihood of obtaining positive results from the IGRA test increases with age.This implies that the accuracy of the predictive model may decline among older individuals.Therefore, for older individuals, it is necessary to exercise extreme caution when applying the model proposed in this study.

In clinical practice, for the diagnosis of TPE, we have the following experience: if a patient is young with fever, increased ratio of mononuclear cells in pleural effusion, elevated concentration of ADA, and positive T-SPOT.TB result in peripheral blood, then the patient's diagnosis is likely to be TPE. However, clinical experience cannot correctly determine the probability of a patient suffering from TPE. Our model not only verified our clinical experience and accurately identified the predictive variables for the diagnosis of TPE but also accurately calculated the probability of a patient with TPE. This improved understanding makes our diagnosis of TPE more accurate.

We strongly recommend validating and updating our prediction model, derived from prospective data, rather than developing new models. In addition, studies that focus on the impact of prediction models on clinical practice, preferably through the clinical decision support system test, are also an important last step in implementing prediction models in clinical practice^25^.

## Conclusions

We developed and validated a novel prediction model including age, positive T-SPOT.TB, lnRMMPE and ADAPE ≥ 40 U/L, which could predict the probability of TPE accurately, simply and safely.

## Supplementary Information

Supplementary Figure 1.

Supplementary Figure 2.

Supplementary Table 1.

## Data Availability

The data that support the findings of this study are available from the corresponding author upon reasonable request.
