# Supplementary material for: Development and validation of a prediction model to assess the probability of tuberculous pleural effusion in patients with unexplained pleural effusion
Source: Sci Rep. 2023 Jul 5;13:10904. doi: 10.1038/s41598-023-38048-2 (PMC10322972; doi:10.1038/s41598-023-38048-2)
Supplement: Supplementary file 1 — Supplementary Figure 1. [file 41598_2023_38048_MOESM1_ESM.pdf]

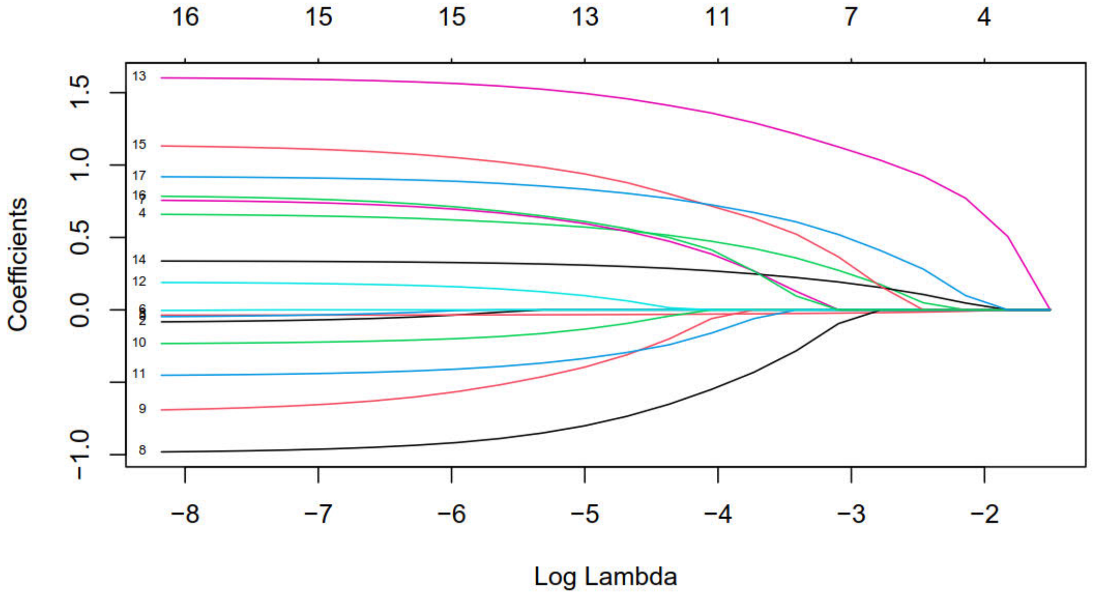

Supplementary Fig.1: LASSO regression analysis for potential predictors selection in the derivation population
