# Supplementary material for: Development and validation of a prediction model to assess the probability of tuberculous pleural effusion in patients with unexplained pleural effusion
Source: Sci Rep. 2023 Jul 5;13:10904. doi: 10.1038/s41598-023-38048-2 (PMC10322972; doi:10.1038/s41598-023-38048-2)
Supplement: Supplementary file 2 — Supplementary Figure 2. [file 41598_2023_38048_MOESM2_ESM.pdf]

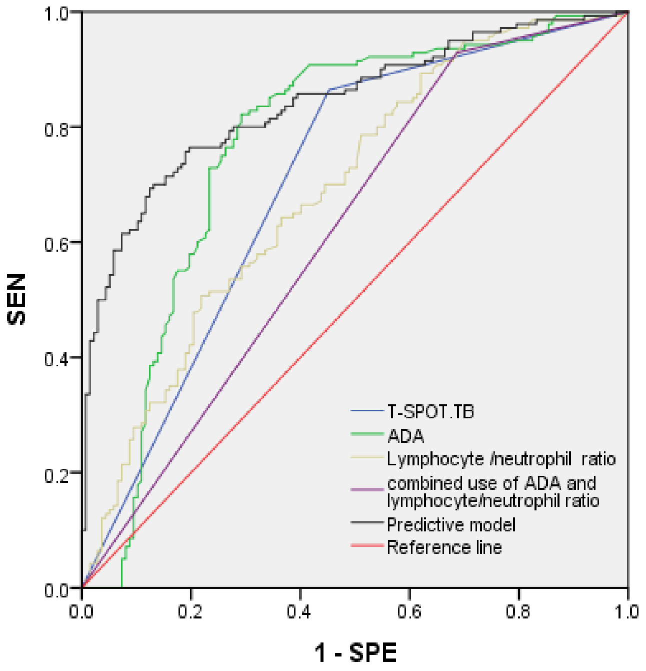

Supplementary Fig.2 ROC for diagnosis of TPE using T-SPOT.TB, ADA, Lymphocyte /neutrophil ratio, combined use of ADA and lymphocyte/neutrophil ratio, and Predictive model.
