# Supplementary material for: Development and validation of a prediction model to assess the probability of tuberculous pleural effusion in patients with unexplained pleural effusion
Source: Sci Rep. 2023 Jul 5;13:10904. doi: 10.1038/s41598-023-38048-2 (PMC10322972; doi:10.1038/s41598-023-38048-2)
Supplement: Supplementary file 3 — Supplementary Table 1. [file 41598_2023_38048_MOESM3_ESM.docx]

Supplementary Table 1 Comparison of diagnostic accuracy for TPE using T-SPOT.TB, ADA, Lymphocyte /neutrophil ratio, combined use of ADA and lymphocyte/neutrophil ratio, and Predictive model.

|  | T-STOP.TB | ADA | Lymphocyte /neutrophil ratio | combined use of ADA ( ＞40 IU/L) and lymphocyte/neutrophil ratio ( ＞0.75) | Predictive model |
| --- | --- | --- | --- | --- | --- |
| Cut-off value |  | 40 IU/L | 0.75 |  | 0.60 |
| AUC  (95% CI) | 0.710  (0.649-0.772) | 0.771  (0.712-0.830) | 0.691  (0.629-0.753) | 0.726  (0.667-0.784) | 0.845 (0.800-0.891) |
| Sensitivity(%) | 86.6 | 46.5 | 91.5 | 41.5 | 70.0 |
| Specificity(%) | 55.4 | 84.9 | 32.4 | 92.1 | 88.0 |
| +PV(%) | 66.5 | 75.9 | 58.0 | 84.3 | 85.3 |
| -PV(%) | 80.2 | 60.8 | 78.9 | 60.7 | 73.9 |
| *P* value^*^ | ＜0.001 | ＜0.05 | ＜0.001 | ＜0.001 |  |

+PV= positive predictive value; -PV=negative predictive value;

* Comparing the AUC of each parameter with the AUC of the predictive model
